# Down-regulation of *c-Met* and *Bcl2* by microRNA-206, activates apoptosis, and inhibits tumor cell proliferation, migration and colony formation

**DOI:** 10.18632/oncotarget.4575

**Published:** 2015-07-25

**Authors:** Chengcao Sun, Zhidong Liu, Shujun Li, Cuili Yang, Ruilin Xue, Yongyong Xi, Liang Wang, Suqing Wang, Qiqiang He, Jie Huang, Songping Xie, Wenyang Jiang, Dejia Li

**Affiliations:** ^1^ Department of Occupational and Environmental Health, School of Public Health, Wuhan University, Wuhan 430071, P. R. China; ^2^ Institute of Global Health, Wuhan University, Wuhan 430071, P. R. China; ^3^ Department of Thoracic Surgery, Beijing Chest Hospital, Capital Medical University, Beijing 101149, P.R. China; ^4^ Wuhan Hospital for the Prevention and Treatment of Occupational Diseases, Wuhan 430071, P. R. China; ^5^ Department of Thoracic Surgery, People's Hospital of Wuhan University, Wuhan 430000, P. R. China

**Keywords:** hsa-miRNA-206(miR-206), c-Met, Bcl2, non-small cell lung cancer (NSCLS), proliferation

## Abstract

Hsa-miRNA-206 (miR-206), highly expressed in skeletal muscle, has recently been discovered to have anticancer properties in different tissues. However, the role of miR-206 on lung cancer is still ambiguous. In this study, we investigated the role of miR-206 on the development of lung cancer. The results indicated that miR-206 expression was suppressed in lung cancer tissues and very low levels were found in non-small cell lung cancer (NSCLS) cell liness. Transient transfection of miR-206 into cultured A549 and SK-MES-1 cells led to significant decrease in cell growth, migration, invasion and colony formation, and promoted cell apoptosis. Using bioinformatics, we identified putative miR-206 binding sites within the 3′-untranslated region (3′-UTR) of the human c-Met and Bcl2 mRNA. The expression of c-Met and Bcl2 proteins were shown to be down-regulated after treated with miR-206 by subsequent Western blot and qRT-PCR analysis. Conversely, up-regulation of c-Met and Bcl2 were confirmed in tissue samples of human lung cancer, with its level inversely correlated with miR-206 expression. In addition, miR-206 also decreased the gene expression of MMP-9, CCND1 and CCND2 while increased the gene expression of p57 (Kip2) in A549 and SK-MES-1 cells. Taken together, our results demonstrated that miR-206 suppressed c-Met and Bcl2 expression in NSCLS and could function as a potent tumor suppressor in c-Met/Bcl2-over expressing tumors. Inhibition of miR-206 function could contribute to aberrant cell proliferation, migration, invasion and apoptosis, leading to NSCLS development.

## INTRODUCTION

Lung cancer is the leading cause of cancer-related deaths both in men and women around the world for several decades. There are estimated to be 1.8 million new cases in 2012 (12.9% of the total), killing about 1.59 million (19.4% of the total) people per year globally, extrapolating from a 2012 International Agency for Research on Cancer (IARC) risk assessment [1]. Approximately 80% of lung cancers are classified histopathologically as non-small cell lung carcinomas (NSCLS). At early stages of NSCLS, the only treatment is surgery, with a 5-year overall survival rate of 40% [2], whereas chemotherapy is mostly employed for small cell lung cancer (SCLS). These changes are attributed to silencing of tumor suppressor genes, dysregulation of proto-oncogenes, and an up-regulation of genes that promote cell growth and transformation and ultimately tumor development [3].

In recent years, there has been a considerable interest in understanding the role of microRNAs (miRNAs) in disease development, including cancers. MiRNAs, a class of ∼20 – 23 nucleotide (nt) noncoding RNAs, repress the expression of their target genes through binding mRNAs at specific sequences. They control gene expression at post-translational level by various mechanisms, such as decay of mRNAs and blockage of translation [4–6]. The mature miRNA is assembled into a miRNA-induced silencing complex, which then directs its binding to the cognate sequence in the 3′-UTR of target mRNAs. Bioinformatic studies indicate that expressions of 30∼50% of human genes are probably controlled by miRNAs [7, 8]. MiRNAs exhibit enormous regulatory potential, since they can target multiple mRNAs. They regulate gene expression in multiple biological and pathological processes, including cell proliferation, apoptosis, heart disease, neurological disorders and human cancers [10, 11].

Aberrations in expression of miRNAs are associated with different diseases, especially cancers [12, 13]. Gene expression profiling of tumor-specific miRNAs reveal distinct miRNAs signature for many types of cancers, including lung cancer [14, 15]. Several miRNAs deregulated in human malignancies exhibit oncogenic or tumor suppressor properties, depending upon the cell type [13]. Some miRNAs, such as let-7, miR-145 and miR-29a/b/c, are down-regulated in lung cancer [16–18]. In fact, let-7 exhibited growth-inhibitory properties in lung cancer cells [17]. Thus, it is conceivable that more miRNAs will play a critical role in lung tumorigenesis and can potentially serve as biomarkers and targets for anticancer therapy.

MiRNA-206 is a skeletal muscle-enriched miRNA that inhibits proliferation of progenitor cells and promotes myogenesis [19, 20]. However, further studies showed miR-206 is expressed in other tissues and functioned as a potential anticancer role [21]. Kondo *et al* found that miR-206 is down-regulated in breast cancer and represses estrogen receptor alpha (ERα) expression [22]. These authors proposed that loss of miR-206 may be linked with breast cancer development. Another study indicated that miR-206 levels are low in melanoma tumors compared with normal skin samples, and it also induces G1 arrest in melanoma cell lines [23]. MiR-206 has also been shown to function as a pro-apoptotic factor in HeLa cells by targeting Notch3 signaling [24]. All these studies further implicate a tumor suppressor role for miR-206.

In this study, we show for the first time that miR-206 directly targets and regulates the full-length 3′-UTR of the human BCL2 (B-cell lymphoma-2) gene, and confirmed that miR-206 directly targets and regulates the full-length 3′-UTR of the human MET mRNA, which are up-regulated in many cancers, including lung cancer. c-Met is encoded by MET gene, and plays a key role in the control of invasive growth not only during tumorigenesis but also in embryonic development, organ development, and inflammatory response [25]. Bcl-2, encoded by anti-apoptosis gene BCL2, is over expressed and inhibits cell apoptosis in lung tumor tissues. Here, we reported that miR-206 is indeed suppressed in primary lung cancers compared with the matching normal tissues, and found 3′-UTR of the human MET and BCL2 mRNA are really targets of miR-206. Collectively, we discovered that miR-206 inhibits non-small cell lung cancer A549 ang SK-MES-1 cell growth, migration, invasion and colony formation, and promoted cell apoptosis by targeting 3′-UTR of c-Met and Bcl2.

## MATERIALS AND METHODS

### Tissue collection

Lung cancer tissues and normal tissues were obtained from patients who had undergone surgery at the People's Hospital of Wuhan University, between 2013 and 2015 and who were diagnosed with lung cancer based on histopathological evaluation. No local or systemic treatment had been conducted in these patients before the operation. All the tissue samples were collected, immediately snap frozen in liquid nitrogen, and stored at −80°C until RNA extraction. The study was approved by the Research Ethics Committee of Wuhan University (Wuhan, Hubei, PR China). Informed consent was obtained from all patients.

### Cell culture and transfection

The human non-small cell lung cancer cell line, A549 and SK-MES-1, were grown in RPMI 1640 or DMEM medium (Gibco, USA) containing 10% heat-inactivated (56°C, 30 min) fetal calf serum, 2 mmol/L glutamine, penicillin (100 U/mL) and streptomycin (100 U/mL), which was maintained in an incubator at 37°C with 5% CO_2_ in a humidified atmosphere. Has-miRNA-206 mimic and mimic negative control, has-miRNA-206 inhibitor and inhibitor negative control were purchased from RiboBio Co., Ltd. (Guangzhou, China). For convenience, has-miRNA-206 mimic and mimic negative control, has-miRNA-206 inhibitor and inhibitor negative control were simply referred to as miR-206 mimic and miR mimic NC, miR-206 inhibitor and miR inhibitor NC, respectively. Complete medium without antibiotics was used to culture the cells at least twenty four hours prior to transfection. The cells were washed with 1× PBS (pH7.4) and then transiently transfected with 50 nM miR-206 mimic or miR mimic NC, 100 nM miR-206 inhibitor or miR inhibitor NC, using Lipofectamine™ 2000 (Invitrogen, Carlsbad, CA, USA) according to the manufacturer's instructions.

### Western blot analysis

Forty eight hours after transfection, total protein was extracted from the A549 cells using RIPA cell lysis reagent containing proteinase and phosphatase inhibitors (Solarbio) at 4°C for 30 min. Cell lysates were centrifuged at 12,000 × g for 20 min at 4°C, and the protein concentrations of the supernatant were determined using the BCA protein assay reagent kit (Beyotime). The supernatants containing total protein were then mixed with a corresponding volume of 5× SDS loading buffer and heated at 100°C for 10 min. Then, the supernatant lysates were run on 10% SDS-polyacrylamide gels (50 μg/lane), and proteins were transferred to poly (vinylidene fluoride) (PVDF) membranes (Hertfordshire, UK) by semidry electroblotting (1.5 mA/cm2). PVDF membranes were then incubated in blocking buffer [Tris-buffered saline (TBS) supplemented with 0.05% (vol/vol) Tween 20; TBST] containing 5% (wt/vol) skimmed milk powder for 120 min at room temperature followed by three 10 min washes in TBST. The PVDF membranes were then incubated with anti-c-Met (1:1000 dilutions, Affinity), anti-Bcl2 (1:1,000 dilutions, Affinity), anti-cyclin D1 (1:1,000 dilutions, Affinity) and anti-GADPH (1:5,000 dilutions, Affinity) as internal normalizers in TBST containing 5% (wt/vol) skimmed milk powder (antibody buffer) overnight at 4°C on a three-dimensional rocking table. Then the membranes were washed three times for 10 min in TBST and then incubated with goat anti-rabbit IgG conjugated to horseradish peroxidase (1:12,000 dilutions) in antibody buffer for 120 min. Finally, membranes were washed three times for 10 min in TBST and exposed to ECL Advance reagent (GE Healthcare Biosciences, Buckinghamshire, UK) for 2 min as described in the manufacturer's protocol. Then membranes were exposed to Hyperfilm-ECL (GE Healthcare Bio-Sciences) for 2–5 min and visualized using a Fluor S Multimager and Quantity One 4.1 (Bio-Rad Laboratories, Hercules, CA ). The molecular weights of the bands were calculated by a comparison with prestained molecular weight markers (molecular weight range: 6,500–250,000) that were run in parallel with the samples. Semiquantitative analysis of specific immunolabeled bands was performed using a Fluor S image analyzer and Quantity One 4.1.

### RNA isolation and quantitative reverse transcription poly-merase chain reaction (qRT-PCR)

Total RNA from the cultured cells was extracted using Trizol reagent (Invitrogen) according to the manufacturer's instructions. MiRNA levels were measured by qRT-PCR. For the qRT-PCR detection of mature miR-206 expression, we purchased the Bulge-Loop™ miRNA qRT-PCR Primer Set and the miRNA qRT-PCR Control Primer Set (both from RiboBio). RNA (2 μg) was converted into cDNA using the PrimeScript™ RT reagent kit with gDNA Eraser (Takara, Dalian, China) according to the manufacturer's instructions. qRT-PCR was performed using SYBR^®^ Premix Ex Taq™ II (Takara) in the ABI PRISM^®^ 7300 real-time PCR system (Applied Biosystems, Foster City, CA, USA). GADPH and U6 were used as endogenous controls. In addition, melting curves were used to evaluate non-specific amplification. The relative expression level was calculated using the^2-ΔΔCt^ method. The primer sequences used in this study are presented in Table 1 and 2. The formula and its derivations were obtained from the ABI Prism 7300 sequence detection system user guide. Statistical analysis was performed on the fold change.

**Table 1:** Primer sequences for quantitative reverse transcription (RT)-PCR (miRNA)

miRNA	Sequence (5′ → 3′)
**miR-206**	Reverse-transcribed primer:5′-GTCAGAAGGAATGATGCACAGCCAACAACA-3′
	Forward:5′-CGTCAGAAGGAATGATGCACAG-3′ Reverse:5′-ACCTGCGTAGGTAGTTTCATGT-3′
**U6**	Reverse transcribed Primer:5′-AACGCTTCACGAATTTGCGT-3′
	Forward:5′-CTCGCTTCGGCAGCACA-3′ Reverse:5′-AACGCTTCACGAATTTGCGT-3′

**Table 2:** Primer sequences for quantitative reverse transcription (RT)-PCR(mRNA)

Gene	Sequence (5′ → 3′)
**Human c-Met**	Forward:5′-GTTTCCCAATTTCTGACC-3′ Reverse:5′-TATATCAAAGGTGTTTAC-3′
**Human Bcl-2**	Forward:5′-CTTTGAGTTCGGTGGGGTCA-3′ Reverse:5′-GGGCCGTACAGTTCCACAAA-3′
**Human CyclinD1**	Forward:5′-CTCCTCTCCGGAGCATTTTGATA-3′ Reverse:5′-TTAAAGACAGTTTTTGGGTAATCT-3′
**Human CyclinD2**	Forward:5′-TGCTGTCTGCATGTTCCTGGCCTC-3′ Reverse:5′-ATCTTAGCCAGCAGCTCAGTCAGG-3′
**Human MMP-9**	Forward:5′-CTGCAGTGCCCTGAGGACTA-3′ Reverse:5′-ACTCCTCCCTTTCCTCCAGA-3′
**Human p57**	Forward:5′-CCCTTCTTCTCGCTGTCCTC-3′ Reverse:5′-CTGGTCCTCGGCGTTCA-3′
**Human GAPDH**	Forward:5′-AGCCTTCTCCATGGTGGTGAA-3′ Reverse:5′-ATCACCATCTTCCAGGAGCGA-3′

### Colony formation assay

Cells were transfected with miR-206 mimic or miR mimic NC, miR-206 inhibitor or miR inhibitor NC, as described above. Twenty four hours later, transfected cells were trypsinized, counted and replated at a density of 500 cells/6 cm dish. Ten days later, colonies resulting from the surviving cells were fixed with 3.7% methanol, stained with 0.1% crystal violet and counted. Colonies containing at least 50 cells were scored. Each assay was performed in triplicates.

### Luciferase reporter assays

The 3′-untranslated region (UTR) of human MET and BCL2 were amplified from human genomic DNA and individually inserted into the pmiR-RB-REPORT^TM^ (Ribobio, Guangzhou, China) using the XhoI and NotI sites. Similarly, the fragment of MET or BCL2 3′-UTR mutant was inserted into the pmiR-RB-REPORT^TM^ control vector at the same sites. For reporter assays, A549 and SK-MES-1 cells were co-transfected with wild-type (mutant) reporter plasmid and miR-Ribo^TM^ mimics (miR-Ribo^TM^ negative control) using Lipofectamine 2000 (Invitrogen). Firefly and Renilla luciferase activities were measured in cell lysates using the Dual-Luciferase Reporter Assay system. Luciferase activity was measured forty eight hours post-transfection using dual-glo luciferase reporter system according to the manufacturer's instructions (Promega, Madison, WI, USA). Firefly luciferase units were normalized against Renilla luciferase units to control for transfection efficiency.

### Transwell migration/invasion assay

A549 and SK-MES-1 cells were grown in RPMI 1640 or DMEM medium containing 10% fetal bovine serum to ∼60% confluence and transfected with 50 nM miR-206 mimic or a negative control, 100 nM miR-206 inhibitor or a negative control. After twenty four hours, the cells were harvested by trypsinization and washed once with Hanks' balanced salt solution (Invitrogen). To measure cell migration, 8-mm pore size culture inserts (Transwell; Costar, High Wycombe, UK) were placed into the wells of 24-well culture plates, separating the upper and the lower chambers. In the lower chamber, 500 μL of RPMI 1640 (for A549) or DMEM (for SK-MES-1) containing 10% FBS was added. Then, serum-free medium containing 5 × 10^4^ cells were added to the upper chamber for migration assays, whereas 1 × 10^5^ cells were used for matrigel invasion assays. After twenty four hours of incubation at 37°C with 5% CO_2_, the number of cells that had migrated through the pores was quantified by counting 10 independent visual fields under the microscope (Olympus) using *a* ×20 magnifications, and cell morphology was observed by staining with 0.1% crystal violet. Filters were washed thoroughly with 1 × PBS and dissolved in 500 μL of 33% acetic acid, and absorbance was measured at 570 nm. Absorbance of cells incubated in the serum-free medium in the bottom chamber was used as negative control. Each experiment was performed at least three times.

### BrdU immunofluorescence assay

A549 and SK-MES-1 cells were seeded on sterile cover glasses placed in the 6-well plates. After transfection with miR-206 mimic, miR mimic NC, miR-206 inhibitor, miR inhibitor NC for forty eight hours, the BrdU (5-bromo-2-deoxyuridine; Sigma) stock solution at 10 mg/mL in saline was diluted 1000 × in the culture medium and incubated for 60 min. After washing with PBS, cells were then fixed for 20 min in 4% paraformaldehyde (PFA) and permeabilized with 0.3% Triton X-100 for 10 min. After blocking with 10% goat serum in PBS for 1 h, cells were incubated with a primary rabbit antibody against BrdU (1:200, Abcam) over night at 4°C, and then incubated with the secondary antibody coupled to a fluorescent marker, Cy3, at room temperature for 2 h. After DAPI staining and PBS washing, the cover slips were mounted on to glass slides with anti-fade solution and visualized using a fluorescence microscope (Olympus 600 auto-biochemical analyzer, Tokyo, Japan) with Image-Pro Plus software for image analysis, and 10 microscopic fields were taken for calculating BrdU.

### CCK8 assay

Cell growth was measured using the cell proliferation reagent WST-8 (Roche Biochemicals, Mannheim, Germany). After plating cells in 96-well microtiter plates (Corning Costar, Corning, NY) at 1.0 × 10^3^ /well, 10 μL of CCK8 was added to each well at the time of harvest, according to the manufacturer's instructions. One hour after adding CCK8, cellular viability was determined by measuring the absorbance of the converted dye at 450 nm.

### Hoechst 33342 Staining

Apoptotic morphological changes in the nuclear chromatin of cells were detected by Hoechst 33342 staining. A549 and SK-MES-1 cells were seeded on sterile cover glasses placed in the 6-well plates. After overnight growth, cells were transfected with miR-206 mimic, miR mimic NC, miR-206 inhibitor and miR inhibitor NC for forty eight hours. Then cells were washed with phosphate-buffered saline (PBS) and fixed with 4% PFA for 10 min, and then incubated with 5 ug/mL Hoechst 33342 staining solution for 10 min. After three washes with PBS, the cells were viewed and recorded by Image-Pro Plus software (Media Cybernetics, Silver Spring, MD) under a fluorescence microscope (Olympus 600 auto-biochemical analyzer, Tokyo, Japan).

### Annexin V-FITC/PI analysis

A549 cells were seeded on sterile cover glasses placed in the 6-well plates. After overnight growth, cells were transfected with miR-206 mimic, miR mimic NC, miR-206 inhibitor and miR inhibitor NC for forty eight hours. Then cells were washed with phosphate-buffered saline (PBS) and fixed with 4% PFA for 10 min, and then incubated with Annexin V-FITC and Propidium (BestBio, Shanghai, China) following the manufacturer's instructions. Then they were finally observed under fluorescence microscopy (Olympus 600 auto-biochemical analyzer, Tokyo, Japan). Using Image-Pro Plus software (Media Cybernetics, Silver Spring, MD) to record images and analyze cell apoptosis.

### Flow cytometry

A549 cells transfected with miR-206 mimic or negative control were trypsinized and resuspended in 1 × binding buffer at 1 × 10^6^ cells/mL. 100 μL of this cell suspension was incubated with 5 μL of FITC-Annexin V and 5 μL propridium iodide (PI) for 15 minutes in the dark. The reaction was terminated with the addition of 400 μL 1 × binding buffer and analyzed with (FACSCalibur using the CellQuest software (Becton Dickinson). FITC-Annexin V-positive and PI-negative cells were considered as apoptotic and the experiments were carried out in triplicates.

### Wound healing assay *in vitro*

The A549 and SK-MES-1 cells were seeded in 6-well plates and incubated for twenty four hours, a linear wound was tehncreated by dragging a 1-mL pipette tip through the monolayer prior to transfection. Cellular debris was removed by gentle washes with culture medium, following which transfection was performed immediately, and the cells were allowed to migrate for a further forty eight hours. The healing process was dynamically photographed after the wound was introduced using a microscope (Olympus 600 auto-biochemical analyzer, Tokyo, Japan). Migration distance was measured from images (5 fields) taken at each indicated time point. The gap size was analyzed using Image-Pro Plus 6.0 software. The residual gap between the migrating cells from the opposing wound edge was expressed as a percentage of the initial gap size.

### Caspase-3 activity assay

The activity of caspase-3 was determined using the Caspase-3 activity kit (Beyotime Institute of Biotechnology, Haimen, China). To evaluate the activity of caspase-3, cell lysates were prepared after their respective treatment with various designated treatments. Assays were performed on 96-well microtitre plates by incubating 10 μL protein of cell lysate per sample in 80 μL reaction buffer (1% NP-40, 20 mM Tris-HCl (pH 7.5), 137 mM Nad and 10% glycerol) containing 10 μL caspase-3 substrate (Ac-DEVD-pNA) (2 mM). Lysates were incubated at 37°C for 4 h. Samples were measured with an ELISA reader at an absorbance of 405 nm. The detail analysis procedure was described in the manufacturer's protocol.

### Transfection of siRNA

Three siRNA duplexes targeting human MET (GenBank accession no. NM_000245) or BCL2 mRNA (GenBank accession no. NM_000633) were designed and synthesized by Guangzhou RiboBio Company (Guangzhou, China). For transfection, the cells were plated on an antibiotic-free growth medium at 60% confluence approximately twenty four hours before transfection. RNA oligonucleotides were transfected at a final concentration of 50 nM, using Lipofectamine 2000 (Invitrogen, USA) according to the manufacturer's protocol.

### Tumor formation in BALB/c nude mice

BALB/c athymic nude mice (male, 4–6-weeks old and 16–20 g) were purchased from Hubei Research Center of Laboratory Animal (Wuhan, China). All animal experiments were carried out in accordance with the Guide for the Care and Use of Laboratory Animals of Wuhan University. To establish lung cancer xenograft model, 5 × 10^6^ A549 cells were suspended in 100 μL phosphate-buffered saline and inoculated subcutaneously into the flanks of nude mice. After 8 days, the transplanted nude mice were randomly divided into two groups (*n* = 6 each). miR-206 agomir (miR-206) or miR agomir NC (NC) (RiboBio Co., Ltd, Guangzhou, China) was directly injected into the implanted tumor at the dose of 1 nmol (in 20 μL phosphate-buffered saline) per mouse every 4 days for seven times. The tumor size was monitored by measuring the length (L) and width (W) with calipers every 4 day, and the volumes were calculated using the formula: (L × W^2^)/2. Mice were killed by cervical dislocation after anaesthetized with 10% chloral hydrate in day 36, and the tumors were excised and snap-frozen for protein and RNA extraction.

### Statistical analysis

All experiments were repeated 3 times independently. The results are presented as the means ± standard error mean (SEM). A two-tailed paired *t*-test was performed using SPSS 19.0 software in order to detect significant differences in measured variables between groups. A value of *P* < 0.05 was considered to indicate a statistically significant difference.

## RESULTS

### MiR-206 is down-regulated in primary human lung cancer

**Figure 1:**
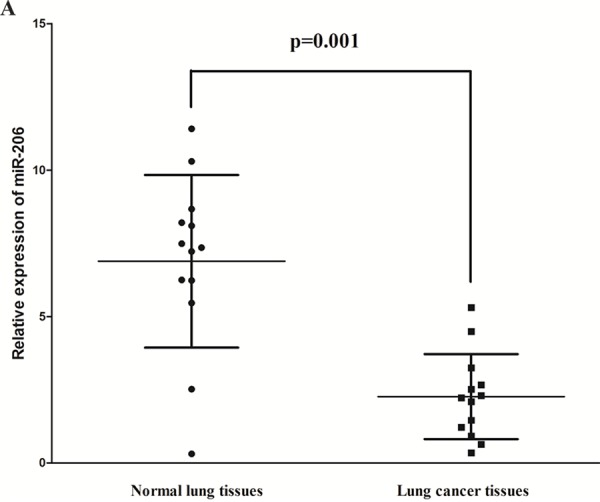

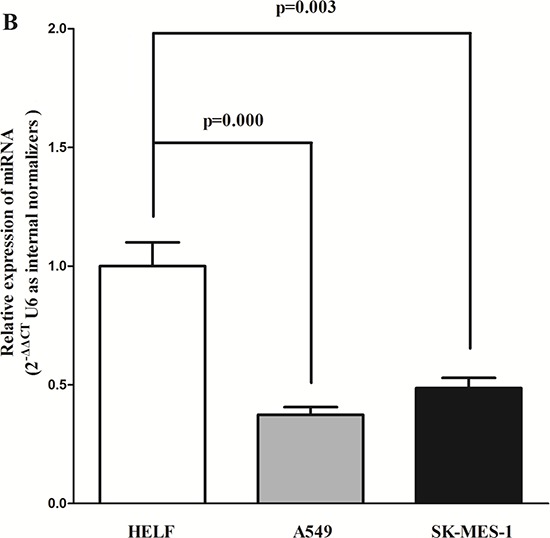
Expression miR-206 is significantly down-regulated in primary human lung cancer **A.** miR-206 is significantly decreased in primary human lung cancer tissues in comparison to matched-normal lung cancer tissues. *n* = 13 for each group. **B.** The expression level of miR-206 in two NSCLS cell lines and normal HELF cells. Assays were performed in triplicate. Means ± SEM are shown. Statistical analysis was conducted using student *t*-test.

To determine whether miR-206 is down-regulated in lung cancer, we measured the mature miR-206 level in human primary lung tumors (NSCLS) and pair-matched lung tissues by qRT-PCR. We used U6 that is not deregulated in lung cancer for normalization. The results showed that miR-206 expression in the tumors was significantly (*p* < 0.05) reduced in 13 lung cancers relative to their matched controls among 13 samples analyzed (Fig. 1A). Next, we examined miR-206 expression in NSCLS cell lines, and results demonstrated a lower expression of miR-206 in A549 and SK-MES-1 cell lines, compared with that of in normal lung cells HELF (Fig. 1B). Thus, it was concluded that the decreased expression of miR-206 might play an important role in lung cancer progression and development.

### Expression of c-Met, Bcl2 and cyclin D1 are up-regulated in primary human lung cancer

**Figure 2:**
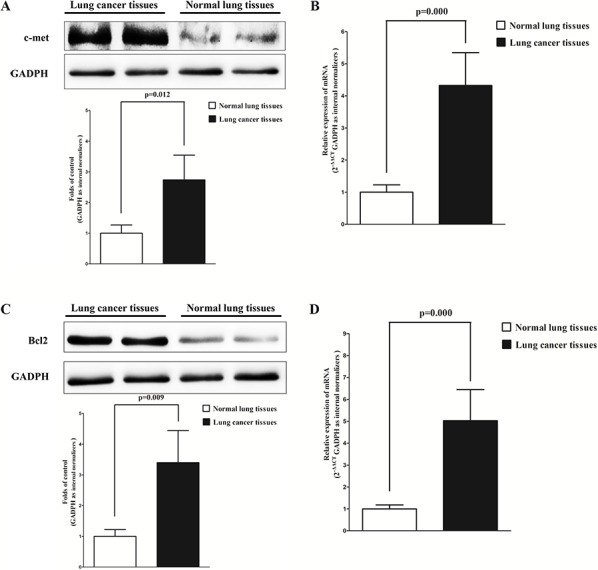

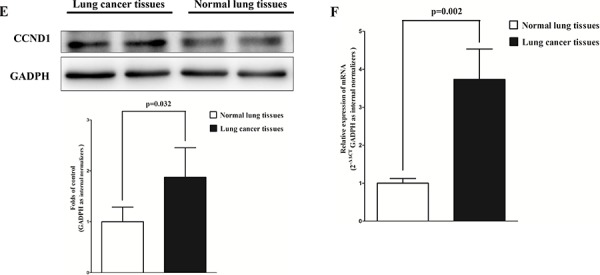
c-Met, Bcl2 and cyclin D1 are up-regulated in primary lung cancer **A.** Western-blot of c-Met protein in lung cancer tissues and normal lung cancers. **B.** qRT-PCR of c-Met mRNA in lung cancer tissues and normal lung cancers. **C.** Western-blot of Bcl2 protein in lung cancer tissues and normal lung cancers. **D.** qRT-PCR of Bcl2 mRNA in lung cancer tissues and normal lung cancers. **E.** Western-blot of cyclin D1 protein in lung cancer tissues and normal lung cancers. **F.** qRT-PCR of cyclin D1 mRNA in lung cancer tissues and normal lung cancers. *n* = 10 for each group. Means ± SEM are shown. Statistical analysis was conducted using student *t*-test.

c-Met, Bcl2 and cyclin D1 are important oncogene that shown strong power of oncogenicity, by promotion of cell growth, migration, invasion and epithelial mesenchymal transition (EMT), as well as inhibition of cell apoptosis in many tumors including lung cancer [20, 26, 27]. Thus, we next examined c-Met, Bcl2 and cyclin D1 expression in human primary lung tumors (NSCLS) and pair-matched lung tissues, and our western blot results demonstrated that the expression of c-Met, Bcl2 and cyclin D1 proteins were all increased in lung cancer tissues compared with normal lung tissues (Fig. 2A, 2C and 2E). These results were confirmed by qRT-PCR of c-Met, Bcl2 and cyclin D1 mRNA expression (Fig. 2B, 2D and 2F). Since c-Met is the target protein of an important proto-oncogene MET, Bcl2 plays anti-apoptosis role *in vivo* and vitro, and cyclin D1 is the key role on regulation of cell cycle regulated by MET, aberrations of these three proteins might contribute to human lung cancer.

### Silence of MET and BCL2 expression inhibits lung cancer cell growth, migration, invasion and promotes apoptosis

**Figure 3:**
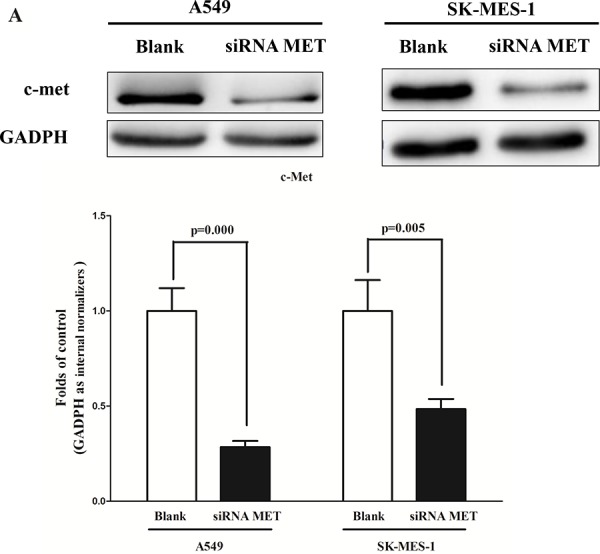

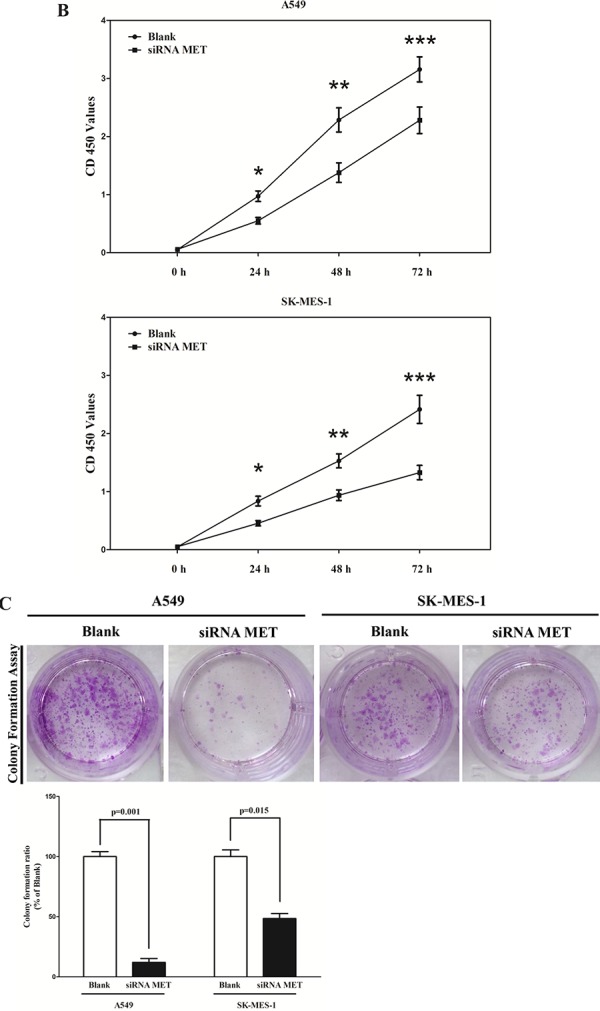

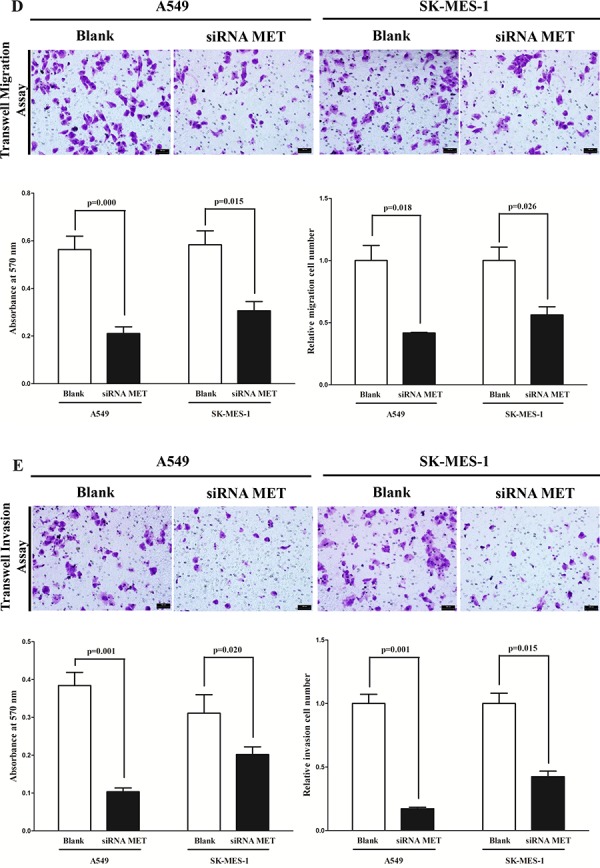

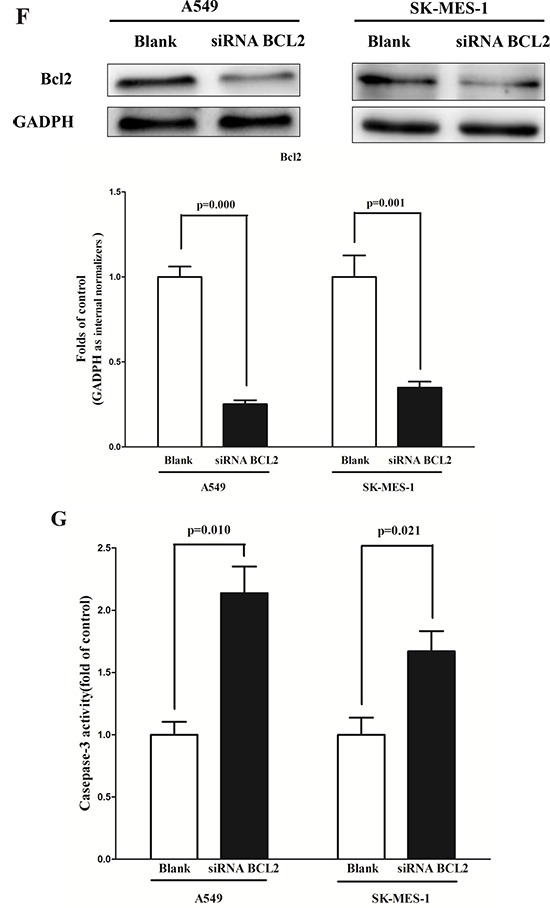
Silence of MET and BCL2 expression inhibits lung cancer cell growth, migration, invasion and apoptosis **A.** Western-blot of c-Met protein in siRNA MET treated and blank A549 and SK-MES-1 cells. **B.** CCK8 assays of A549 and SK-MES-1 cells after transfected (un-transfected) with siRNA MET. **C.** Shown are representative photomicrographs of colony formation assay after transfected with (without) siRNA MET for ten days. **D.** Shown are representative photomicrographs of tanswell migration assay after transfected with (without) siRNA MET. **E.** Shown are representative photomicrographs of tanswell invasion assay after transfected with (without) siRNA MET. **F.** Western-blot of Bcl2 protein in A549 and SK-MES-1 cells treated with (without) siRNA BCL2. **G.** Quantitative representation of casepase-3 activity in A549 and SK-MES-1 cells transfected with (without) siRNA BCL2 for forty eight hours. Assays were performed in triplicate. Means ± SEM are shown. Statistical analysis was conducted using One-way ANOVA.

We next examined the potential tumorigenicity of MET and BCL2 in lung cancer. Silence of MET or BCL2 expression by siRNA (MET and BCL2) significantly inhibited the expression of c-Met (Fig. 3A) or Bcl2 (Fig. 3F), respectively. Moreover, loss of MET expression also contributed to inhibition of lung cancer cell (both A549 and SK-MES-1 cells) growth (Fig. 3B and 3C) and migration (Fig. 3D and 3E). In addition, inhibition of Bcl2 expression promoted apoptosis in lung cancer cell (both A549 and SK-MES-1 cells) (Fig. 3G). These results further verified the powerful tumorigenicity of MET and BCL2 in lung cancer. Thus, we adopted MET and BCL2 for as targeted oncogenes.

### MiR-206 targets human MET and BCL2

**Figure 4:**
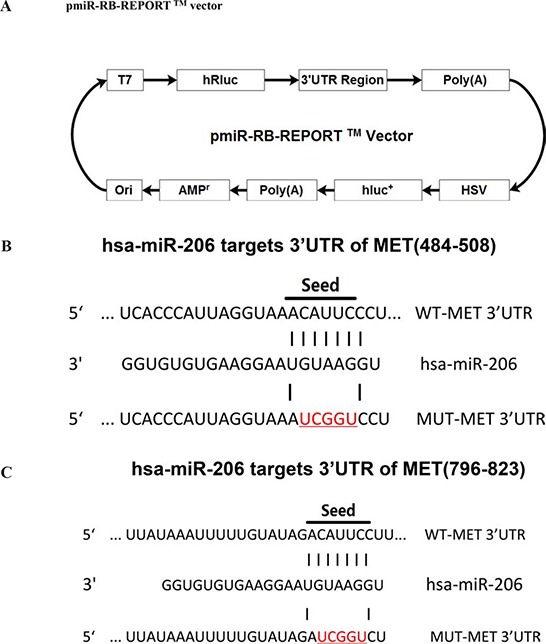

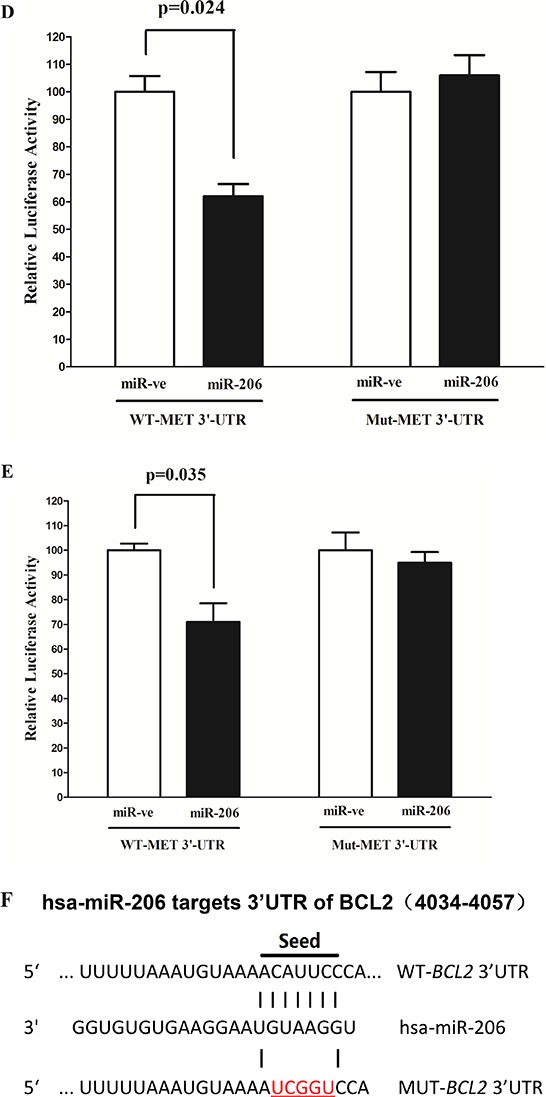

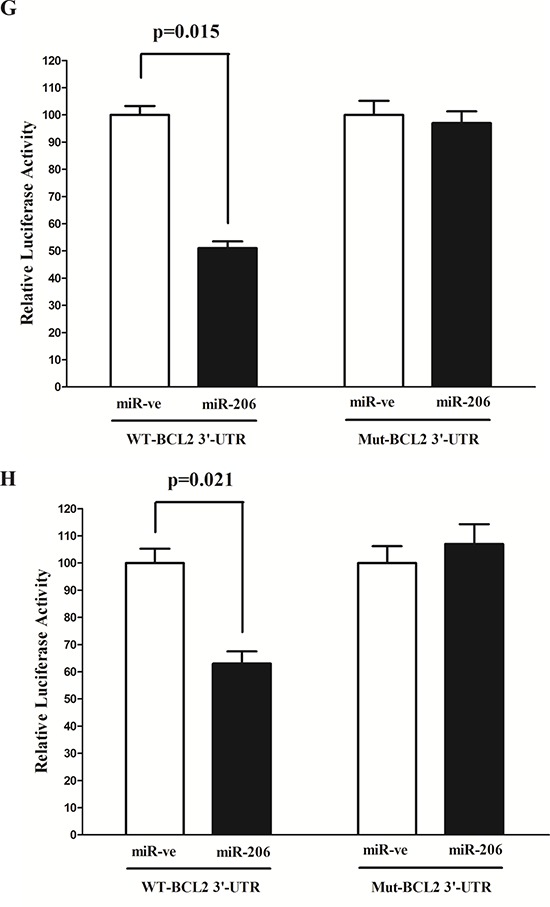
MET and BCL2 proto-oncogene are targets of miR-206 at specific 3′-UTR site **A.** pmiR-RB-REPORT TM dualluciferase reporter vector. **B** and **C.** The 3′-UTR of MET harbors two miR-206 cognate sites. **D-E.** Relative luciferase activity of reporter plasmids carrying wild-type or mutant BCL2 3′-UTR in A549 and SK-MES-1 cells co-transfected with negative control (NC) or miR-206 mimic. **F.** The 3′-UTR of BCL2 harbors one miR-206 cognate site. **G-H.** Relative luciferase activity of reporter plasmids carrying wild-type or mutant BCL2 3′-UTR in A549 and SK-MES-1 cells co-transfected with negative control (NC) or miR-206 mimic. Assays were performed in triplicate. Means ± SEM are shown. Statistical analysis was conducted using student *t*-test.

We then explored the underlying molecular mechanism of the antitumorigenic property of miR-206 in lung cancer cells. Since miRNAs primarily mediate their biological functions in animal cells by impeding the expression of target genes, we searched different data bases (TargetScan, microRNA.org and PicTar) for its potential targets that exhibited oncogenic properties. MET (hepatocyte growth factor receptor), which harbors two conserved miR-206 cognate sites, namely, 484–508 and 796–823 of MET 3′-UTR) (Fig. 4B and 4C), is a predicted target of miR-206. Yan *et al* also discovered miR-206 targets the 3′-UTR of c-Met, but they were in 499–505 and 814–820 of MET 3′-UTR [28]. In addition, BCL2 is also a predicted target of miR-206 on account of it harbors one conserved miR-206 cognate site, namely, 4034–4057 of BCL2 3′-UTR (Fig. 4F). To determine whether MET and BCL2 expression are indeed regulated by miR-206, the MET and BCL2 3′-UTR were cloned into a luciferase reporter plasmid (Fig. 4A), and the ability of miR-206 to inhibit expression of the adjacent hRluc coding region was quantified. For this purpose, the luciferase reporter plasmid pmiR-RB-REPORT^TM^-MET-3′-UTR or a mutant reporter plasmids carrying point mutations in the putative miR-206 binding sites was co-transfected with miR-206 mimics or miR mimic NC, separately. The same is done for BCL2. The results show that miR-206 suppresses luciferase activity by approximately 40% in A549 cells and 32% in SK-MES-1 cells when the reporter plasmid carried the wild type MET 3′-UTR (Fig. 4D and 4E), but no significant suppression was observed when the reporter plasmid carried a mutant MET 3′-UTR (i.e., pmiR-RB-REPORT^TM^-mut-MET-3′-UTR). These results suggest that miR-206 binds directly to the predicted binding site(s) in the MET 3′-UTR and negatively regulates MET expression. Moreover, miR-206 suppresses luciferase activity by approximately 50% in A549 cells and 38% in SK-MES-1 cells when the reporter plasmid carried the wild type BCL2 3′-UTR (Fig. 4G and 4H), but no significant suppression was observed when the reporter plasmid carried a mutant BCL2 3′-UTR (i.e., pmiR-RB-REPORT ^TM^-mut-BCL2–3′-UTR). These results suggest that miR-206 binds directly to the predicted binding site(s) in the BCL2 3′-UTR and negatively regulates BCL2 expression.

### MiR-206 inhibits c-Met and Bcl2 in lung cancer cells

**Figure 5:**
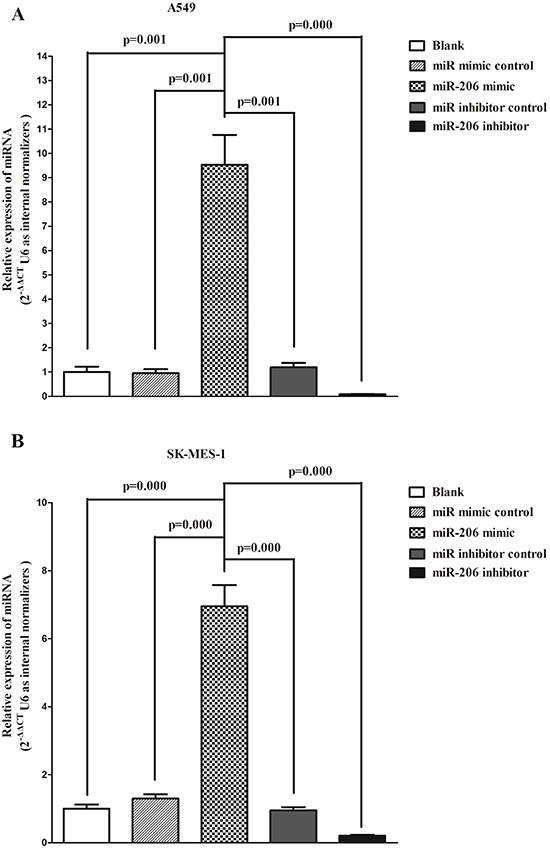

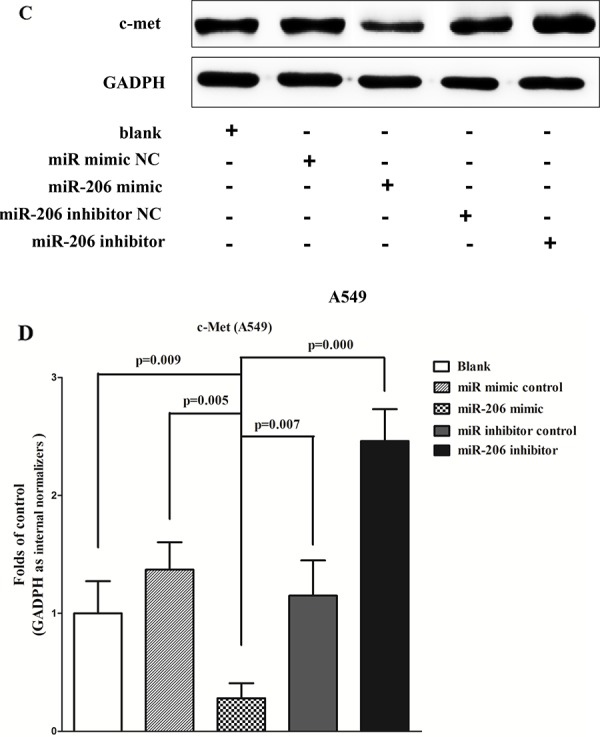

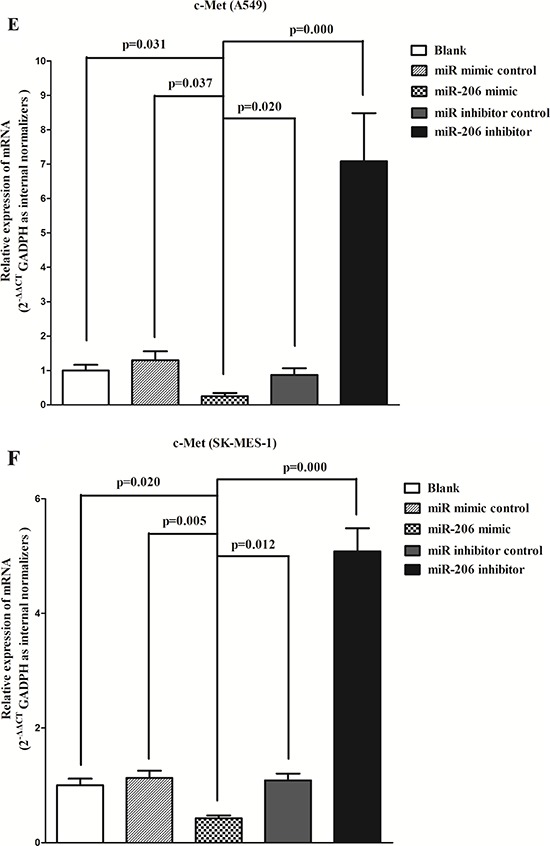

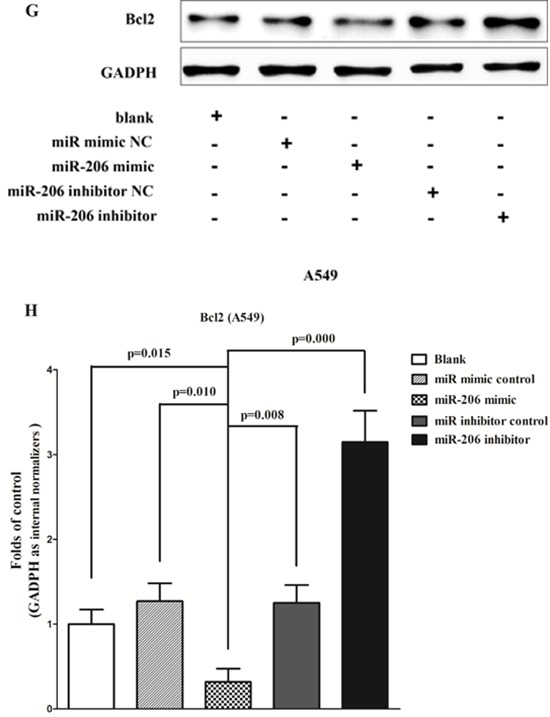

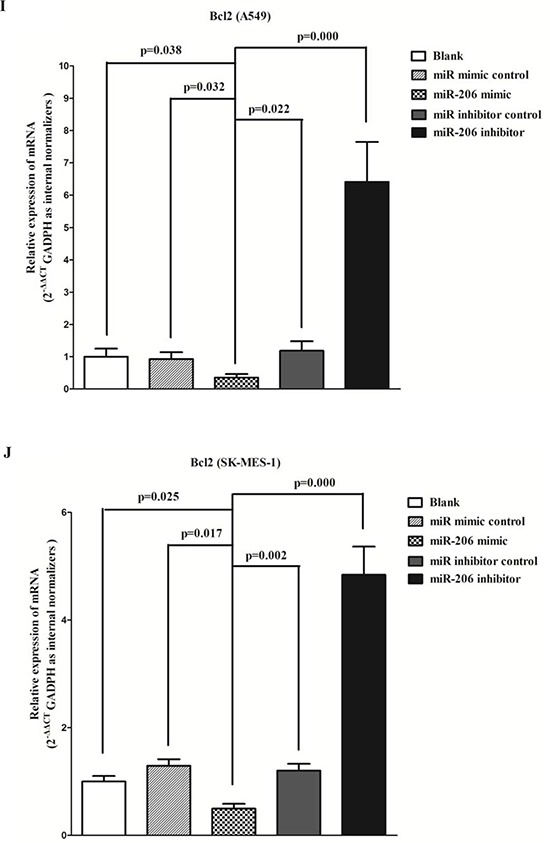

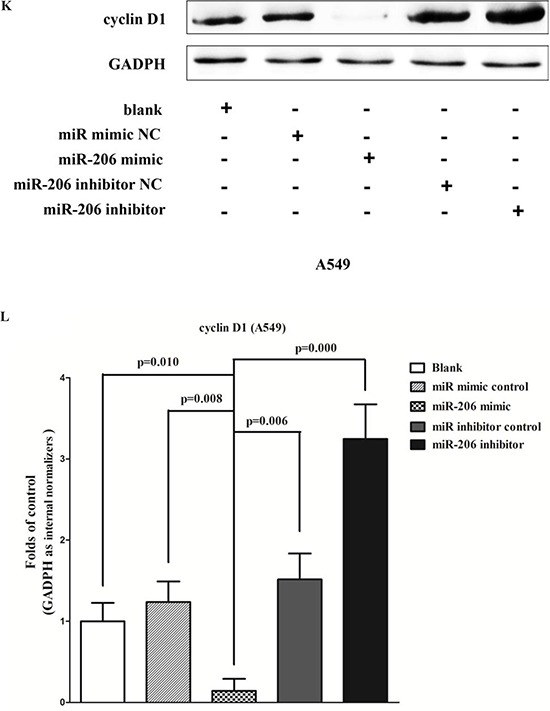

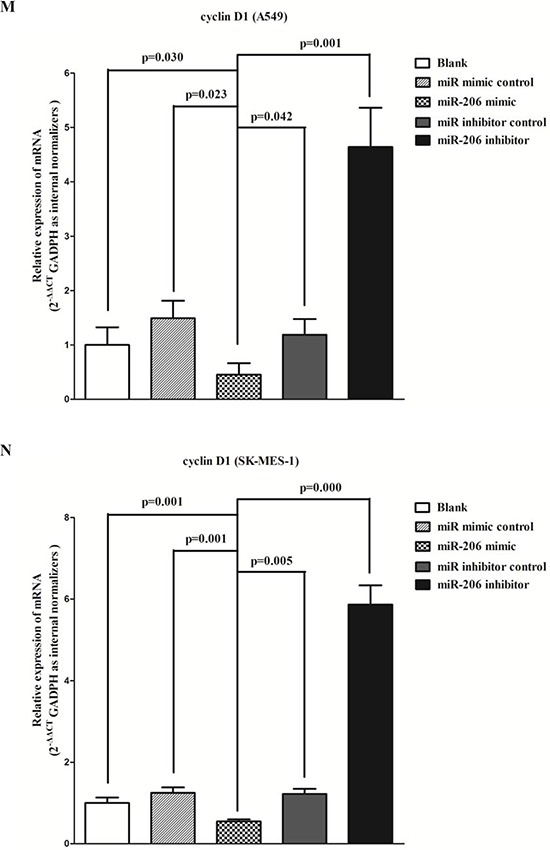

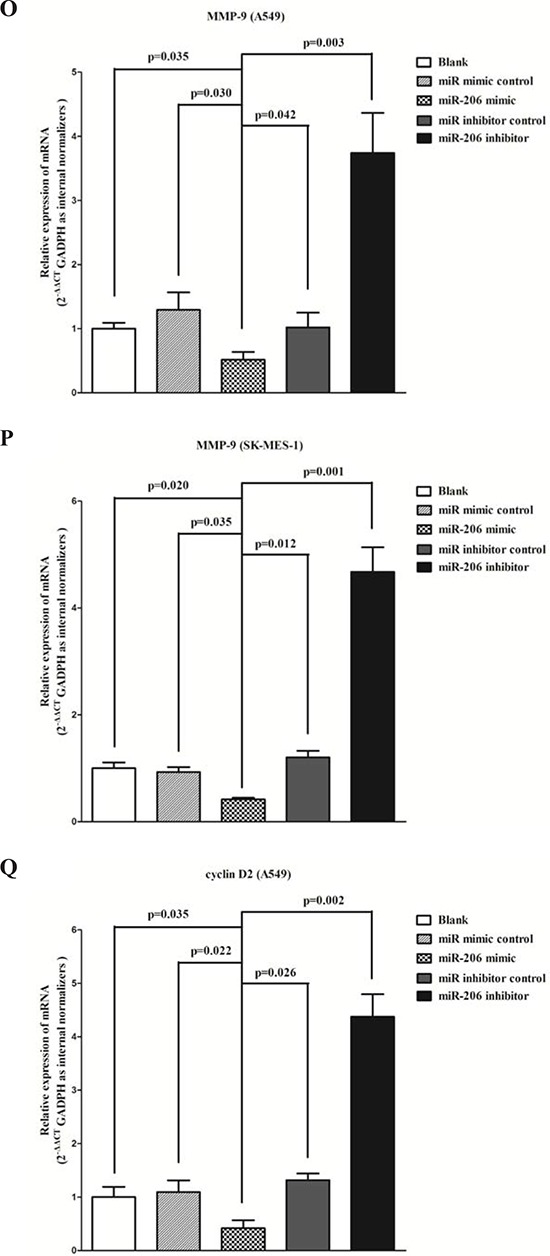

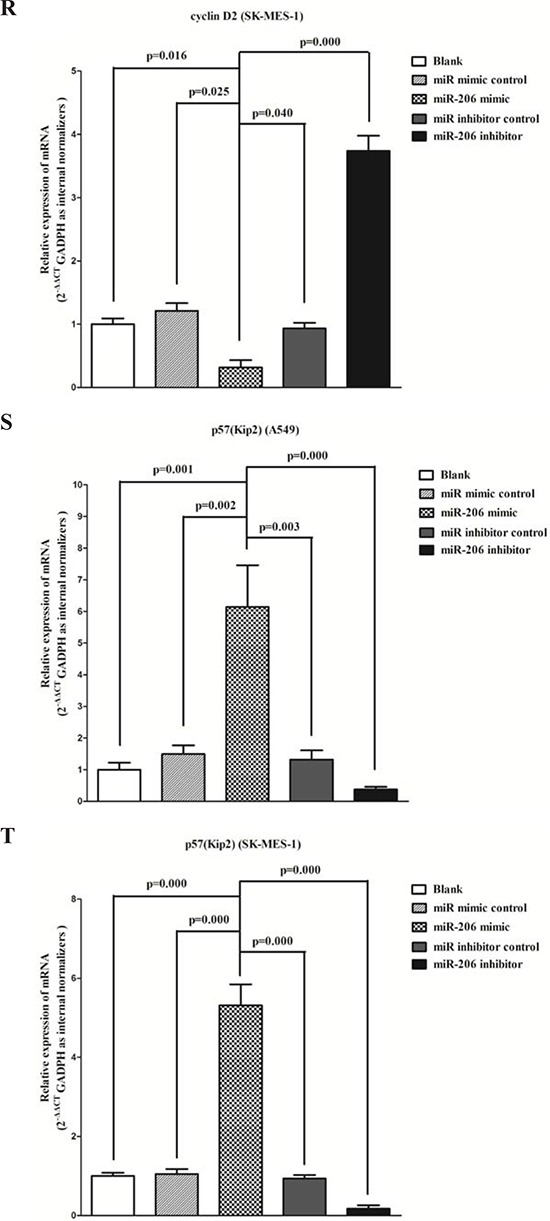
Ectopic expression of miR-206 inhibits c-Met, Bcl2, cyclin D1, cyclin D2 and MMP-9, and inhibits p57 expression in A549 and SK-MES-1 cells **A-B.** miR-206 expression after transfected A549 and SK-MES-1 cells with miR-206 mimic, miR-206 mimic NC, miR-206 inhibitor or miR-206 inhibitor NC for twenty four hours. **C-D.** Expression of c-Met protein in transfected A549 cells. **E-F.** Expression of c-Met, Bcl2 and cyclin D1 mRNA in transfected A549 and SK-MES-1 cells. **G-H.** Expression of Bcl2 protein in transfected A549 cells. **I-J.** Expression of c-Met, Bcl2 and cyclin D1 mRNA in transfected A549 and SK-MES-1 cells. **K-L.** Expression of cyclin D1 protein in transfected A549 cells. **M-N.** Expression of c-Met, Bcl2 and cyclin D1 mRNA in transfected A549 and SK-MES-1 cells. **O-T.** Expression of MMP-9, cyclin D2 and p57 mRNA in transfected A549 and SK-MES-1 cells. Assays were performed in triplicate. Means ± SEM are shown. Statistical analysis was conducted using One-way ANOVA.

To determine whether c-Met and Bcl2 expression are indeed regulated by miR-206 *in vitro*, we transfected NSCLS A549 and SK-MES-1 cells with miR-206 mimic and miR mimic NC. As expected, miR-206 mimic treated cells showed higher expression of miR-206 than blank A549 or SK-MES-1 cells, and miR-206 inhibitor inhibited miR-206 expression (Fig. 5A and 5B). Our western blot results revealed that miR-206 inhibited the expression of c-Met (Fig. 5C and 5D) and Bcl2 (Fig. 5G and 5H) protein in A549 cells, which were confirmed by qRT-PCR of MET (Fig. 5E and 5F) and BCL2 (Fig. 5I and 5J) in A549 and SK-MES-1 cells. However, when treated A549 and SK-MES-1 cells with miR-206 inhibitor, the expression of c-Met (Fig. 5C and 5D) and Bcl2 (Fig. 5G and 5H) protein was remarkably increased in comparison with blank A549 cells, which was confirmed by qRT-PCR of MET (Fig. 5E and 5F) and BCL2 (Fig. 5I and 5J) in A549 and SK-MES-1 cells. These results demonstrated miR-206 inhibited c-Met and Bcl2 expression in A549 cells, and loss of miR-206 would be attributed to the over-expression of c-Met and Bcl2 in lung cancer cells, which were the risks of NSCLS. Moreover, expression of cyclin D1 protein (Fig. 5K and 5L) and mRNA of CCND1 (Fig. 5M and 5N), CCND2 (Fig. 5Q and 5R) and MMP-9 (Fig. 5O and 5P) were also negatively regulated by miR-206, while the pro-apoptosis gene of p57 (Fig. 5S and 5T) was positively regulated by miR-206, in A549 and SK-MES-1 cells. Collectively, the aberration of miR-206 in A549 and SK-MES-1 cells will contribute to the down-regulation of the above key factors related to lung cancer (NSCLS).

### MiR-206 inhibits lung cancer cell proliferation and colony formation

**Figure 6:**
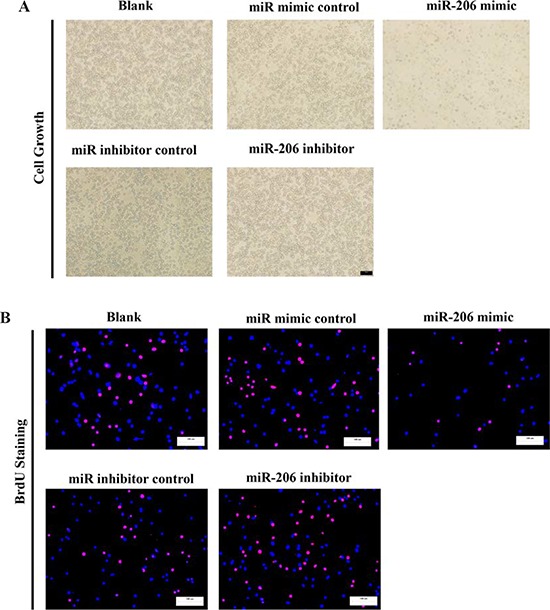

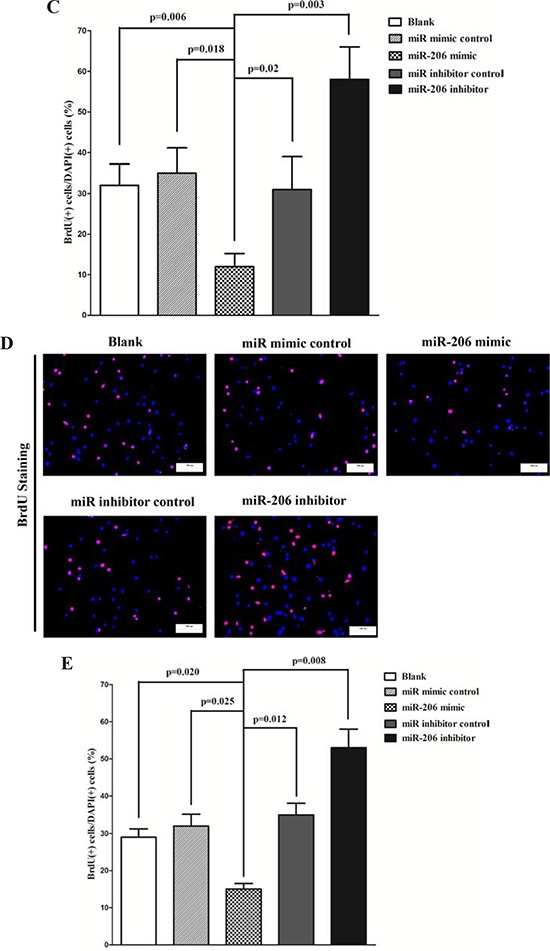

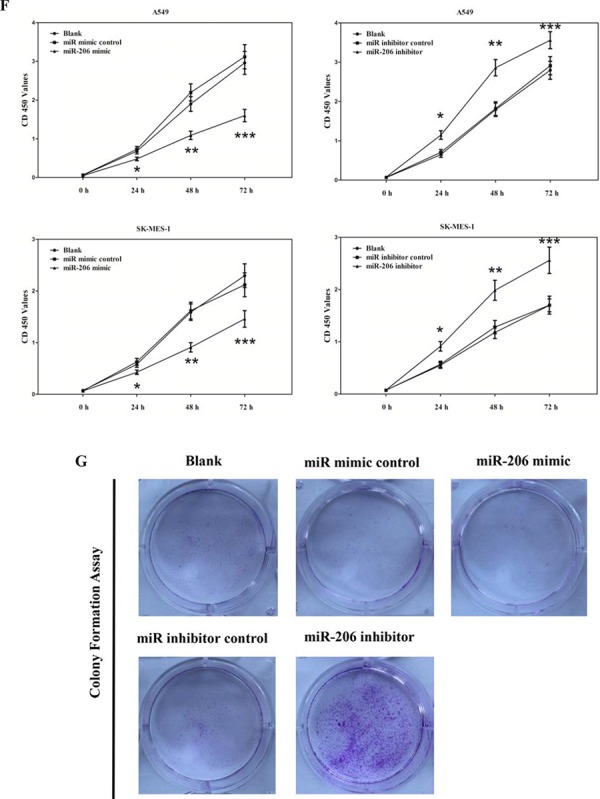

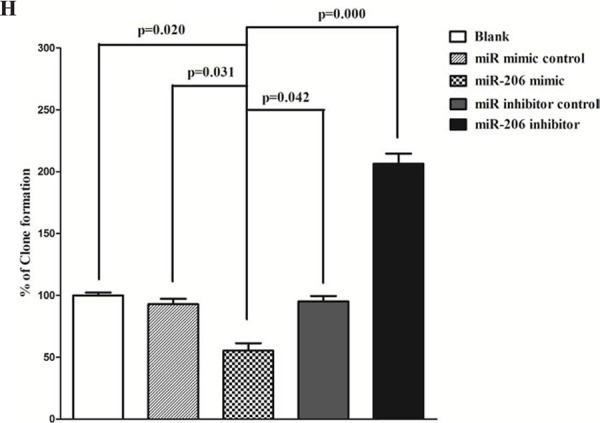
Ectopic expression of miR-206 inhibits proliferation and colony formation of A549 and SK-MES-1 cells **A.** Shown are representative photomicrographs of A549 cells after transfected with miR-206 mimic, miR-206 mimic NC, miR-206 inhibitor ormiR-206 inhibitor NC for forty eight hours. Bar = 100 γm. **B** and **D.** Shown are representative photomicrographs of BrdU staining after transfected A549 and SK-MES-1 cells with miR-206 mimic, miR-206 mimic NC, miR-206 inhibitor or miR-206 inhibitor NC for 24 h. Bar = 100 μm. **C** and **E.** Statistical analysis of BrdU staining. **F.** CCK8 assays of A549 and SK-MES-1 cells after transfected with miR-206 mimic, miR-206 mimicNC, miR-206 inhibitor, miR-206 inhibitor NC. **G.** Shown are representative photomicrographs of colony formation assay after transfected withmiR-206 mimic, miR-206 mimic NC, miR-206 inhibitor or miR-206 inhibitor NC for ten days. **H.** Statistical analysis of colony formationassay. Assays were performed in triplicate. Means ± SEM are shown. Statistical analysis was conducted using One-way ANOVA.

To further investigate the anticancer role of miR-206 in lung cancer, we transfected A549 and SK-MES-1 cells with miR-206 mimic or miR mimic NC, and miR-206 inhibitor or miR-206 inhibitor NC, separately. We first examined the role of miR-206 on A549 and SK-MES-1 cells proliferation. Our results of BrdU staining revealed that miR-206 inhibited A549 and SK-MES-1 cell DNA synthesis by approximately 60% (Fig. 6B and 6C) and 50% (Fig. 6D and 6E), compared with blank A549 and SK-MES-1 cells, respectively. However, miR-206 inhibitor treatment increased A549 and SK-MES-1 cell DNA synthesis by approximately 1.7 folds (Fig. 6B and 6C) and 1.6 folds (Fig. 6D and 6E) compared with blank A549 and SK-MES-1 cells, separately. To verify this result, we also did the CCK8 assay, and results demonstrated that miR-206 over-expression significantly promoted A549 and SK-MES-1 cells vitality, while loss of miR-206 attenuated cell proliferation (Fig. 6F). In addition, we used colony formation assay to investigate the role of miR-206 on clonogenic survival, and results demonstrated miR-206 mimic treatment caused a decrease in the clonogenic survival of A549 cells compared with blank A549 cells (Fig. 6G and 6H), while miR-206 inhibitor-treated A549 cells showed an significant increase in the clonogenic survival, when compared with blank A549 cells (Fig. 6G and 6H).

### MiR-206 inhibits lung cancer cell migration and invasion

Then, we examined the role of miR-206 on A549 and SK-MES-1 cells migration and invasion. Invasion and migration through the basement membrane are characteristics of metastatic cancer cells.

**Figure 7:**
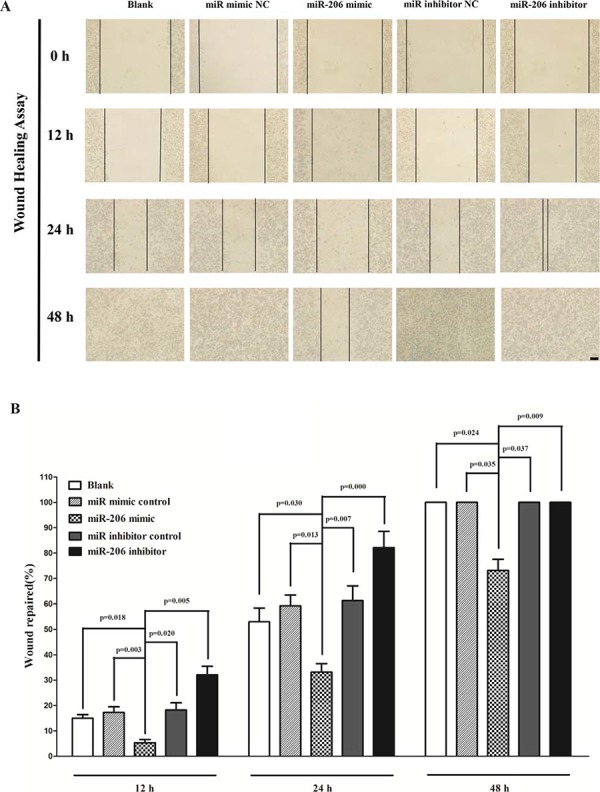

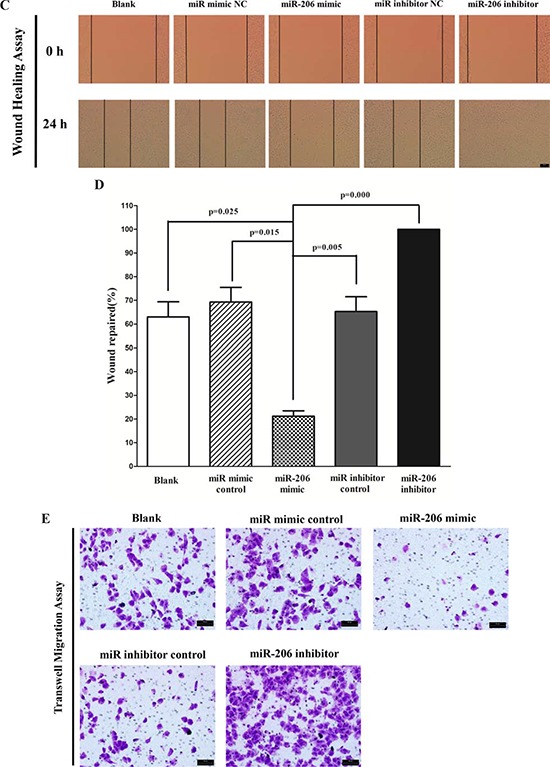

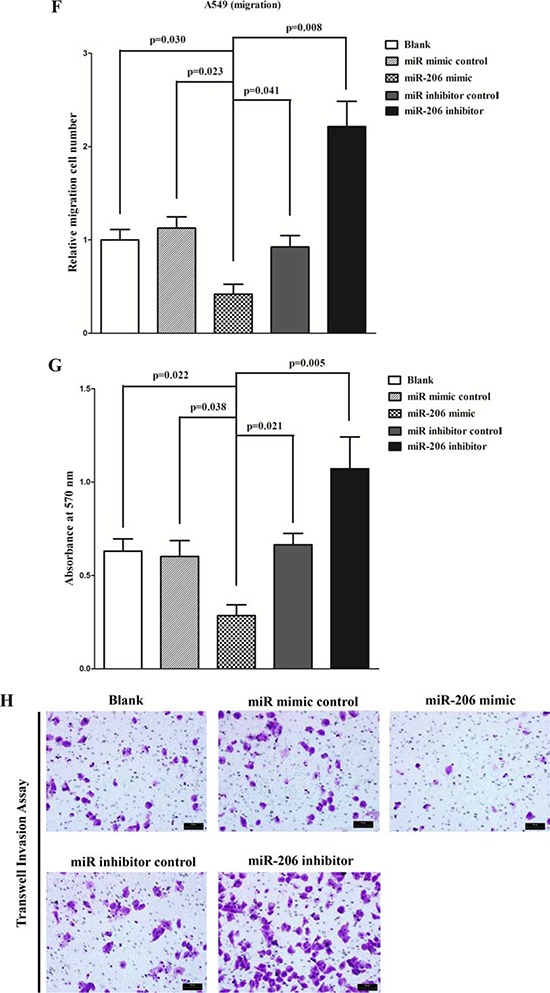

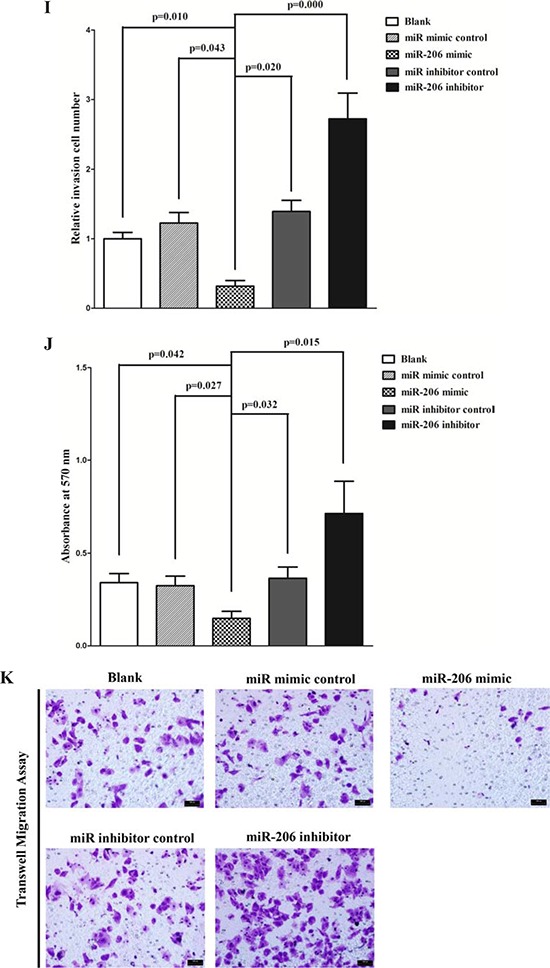

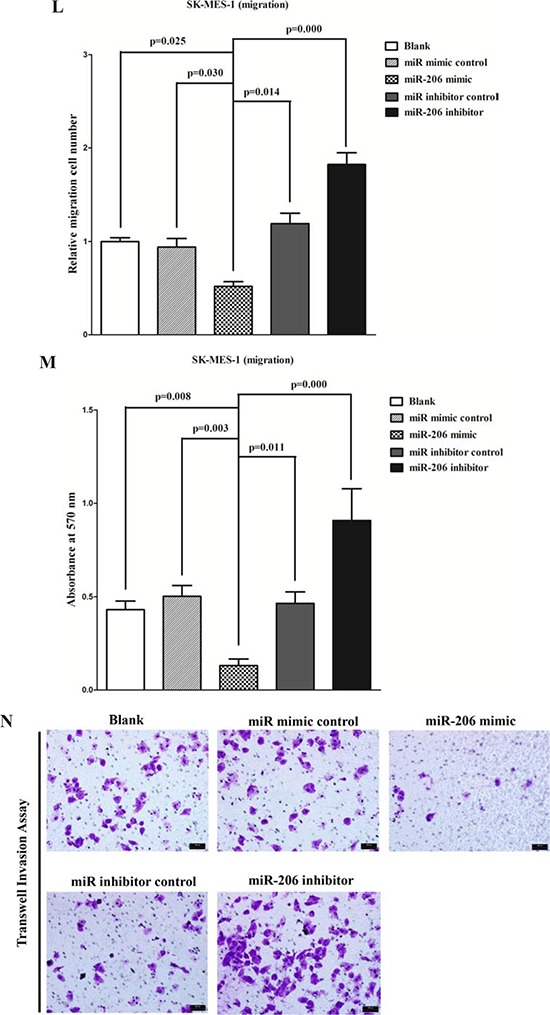

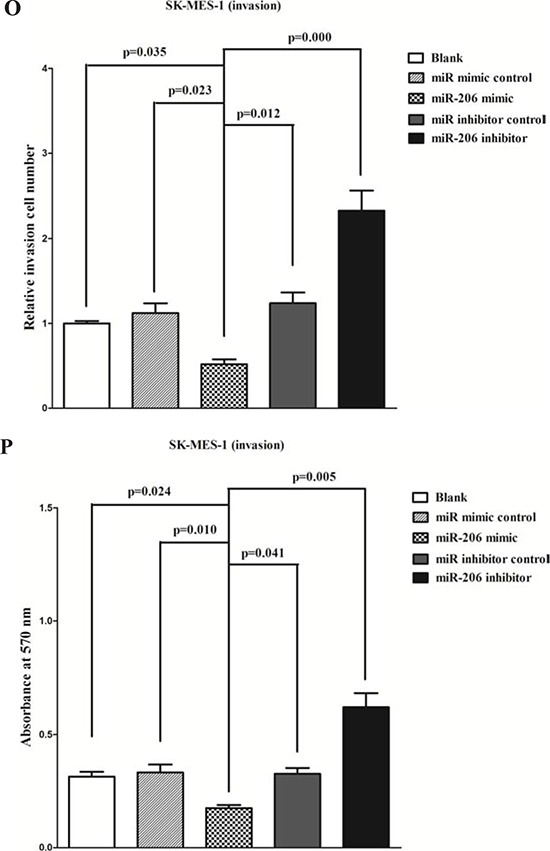
Ectopic expression of miR-206 in A549 and SK-MES-1 cells reduces cell migration and invasion motility **A** and **C.** Shown are representative photomicrographs of “wound healing assay” in A549 and SK-MES-1 cells after transfected miRNAs for twelve hours, twenty four hours and forty eight hours. Bar = 100 μm. **B** and **D.** Statistical analysis of “wound healing assay”. **E** and **K.** A549 and SK-MES-1 cells were loaded onto the top well of a transwell inserts for cell migration assay. After twenty four hours, cells that migrated to the bottom chamber containing serum-supplemented medium were stained with 0.1% crystal violet, visualized under a phase-contrast microscope, and photographed. Bar = 50 μm. **F** and **L.** Total number of cells in five fields was counted manually. **G** and **M**. 0.1% crystal violet-stained cells were solubilized in 33% acetic acid, and absorbance was measured at 570 nm. **H** and **N.** A549 and SK-MES-1 cells were loaded onto the top well of a transwell inserts for cell invasion assay. After twenty four hours, cells that migrated to the bottom chamber containing serum-supplemented medium were stained with 0.1% crystal violet, visualized under a phase-contrast microscope, and photographed. Bar = 50 μm. **I** and **O.** Total number of cells in five fields was counted manually. **J** and **P.** 0.1% crystal violet-stained cells were solubilized in 33% acetic acid, and absorbance was measured at 570 nm. Assays were performed in triplicate. Means ± SEM are shown. Statistical analysis was conducted using One-way ANOVA

We used two different approaches to assess the role of miR-206 on the ability of A549 and SK-MES-1 cells migration. In the first technique, we used a “scratch wound healing” assay. Motility of cells at different time points after generation of the wound was monitored under a microscope. Closure of the wound was complete within forty eight hours in control A549 (Fig. 7A and 7B) and SK-MES-1 cells (Fig. 7C and D). In contrast, miR-206-expressing cells migrated toward the wound at a much slower rate (Fig. 7A-7D). In the second approach, cells were seeded in serum-free medium on the top chamber of a two-chamber trans-well cell culture plate, and the cells migrated to the lower chamber containing complete medium after twenty four hours were photographed (Fig. 7E and 7K) and counted. As expected, migration of miR-206-expressing clones was inhibited by 55% in A549 and 45% in SK-MES-1 cells, compared with the blank A549 and SK-MES-1 cells (Fig. 7E-F and 7K and 7L), respectively. Colorimetric estimation of migrated cells showed a 46% and 62% decrease in miR-206 mimic treated A549 and SK-MES-1 cells, compared with the blank A549 and SK-MES-1 cells (Fig. 7G and M), respectively. However, when treated with miR-206 inhibitor, migration in miR-206-expression defect A549 and SK-MES-1 cells were significantly increased by approximately 4 and 1.8 folds relative to blank A549 and SK-MES-1 cells (Fig. 7E-G, and K-M), respectively.

To investigate the role of miR-206 on A549 and SK-MES-1 cells invasion, we used a transwell invasion assay. As expected, invasion of miR-206-expressing clones was inhibited by 65% in A549 and 47% in SK-MES-1 cells, relative to the blank A549 (Fig. 7H and 7I) and SK-MES-1 cells (Fig. 7N and 7O), respectively. Colorimetric estimation of migrated cells showed a 40% and 35% decrease in miR-206 mimic treated A549 and SK-MES-1 cells, compared with the blank A549 (Fig. 7J) and SK-MES-1 cells (Fig. 7P), respectively. However, when treated with miR-206 inhibitor, invasion in miR-206-expression defect A549 and SK-MES-1 cells were significantly increased by approximately 5 and 2.3 folds relative to blank A549 and SK-MES-1 cells (Fig. 7H-J and N-P), separately.

These results, taken together, clearly demonstrated that miR-206 expression markedly reduces the migration and invasion motility of lung cancer cells.

### MiR-206 promotes lung cancer cell apoptosis

**Figure 8:**
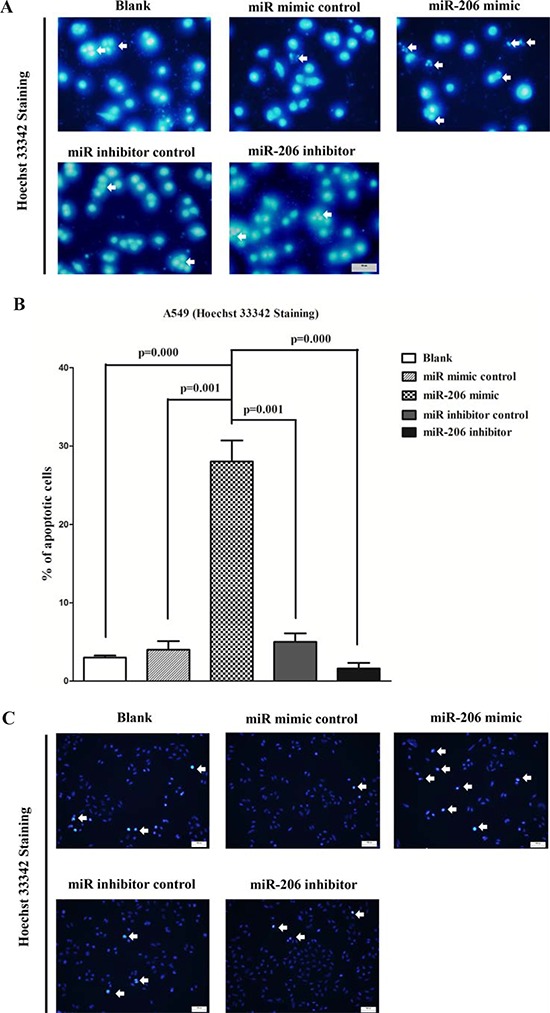

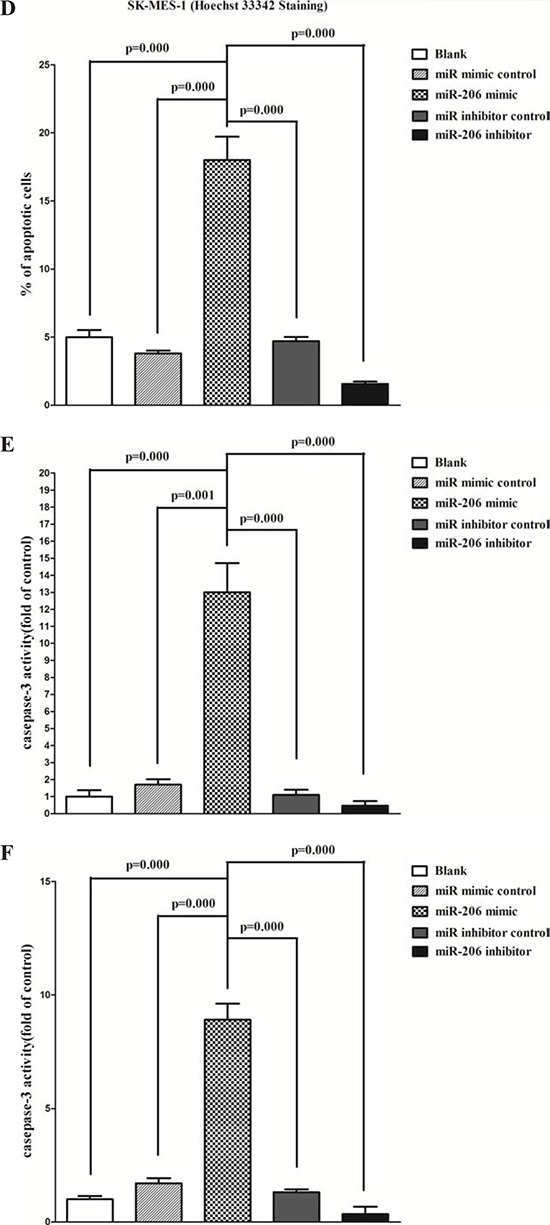

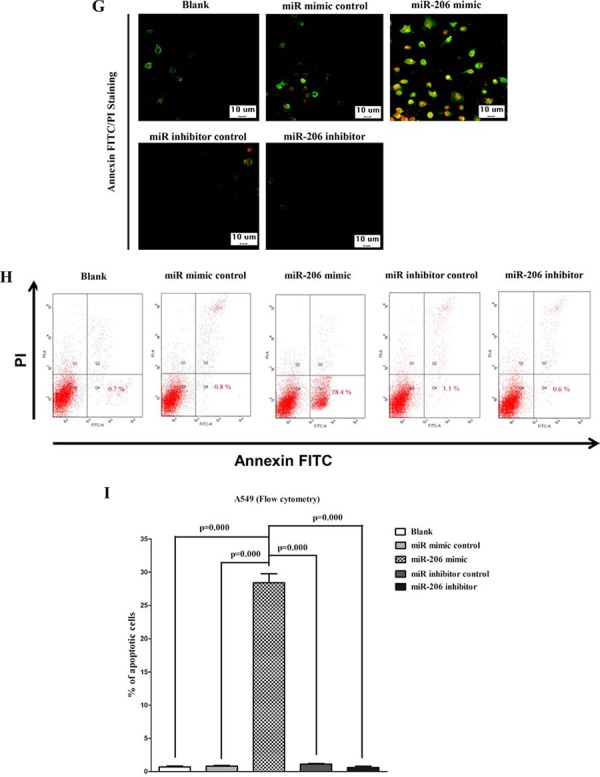
Ectopic expression of miR-206 promotes apoptosis in A549 and SK-MES-1 cells **A** and **C.** Shown are representative photomicrographs of A549 and SK-MES-1 cells stained with Hoechst 33342. Cells grown in coverslips were stained with Hoechst 33342 and photographed under a fluorescence microscope. Cells undergoing DNA fragmentation were counted manually. The arrows denote cells with fragmented nuclei. Bar = 50 μm for A549 cells and Ba*r* = 100 μm for SK-MES-1 cells. **B** and **D.** Quantitative representation of the number of apoptotic after transfecting cells with related miRNAs for forty eight hours. The total number of cells (∼200) with or without fragmented nuclei was counted, and the percentage of apoptotic cells was calculated. **E-F.** Quantitative representation of casepase-3 activity in A549 and SK-MES-1 cells transfected with related miRNAs for forty eight hours. **G.** Shown are representative photomicrographs of cells dual-stained with annexin-FITC/PI. Bar = 10 μm. **H.** Shown are representative photomicrographs of flow cytometric analysis. **I.** Statistical analysis of flow cytometric analysis. Assays were performed in triplicate. Means ± SEM are shown. Statistical analysis was conducted using One-way ANOVA.

Next, we examined the role of miR-206 on A549 and SK-MES-1 cells apoptosis. Hoechst 33342 staining revealed that miR-206 increased the number of apoptosis cells in A549 and SK-MES-1 cells, while inhibition of miR-206 attenuated DNA damaging and nuclear fragmentation (Fig. 8A-8D), separately. Moreover, we tested the caspase-3 activity after treatment of A549 and SK-MES-1 cells with miR-206 mimic or miR-206 mimic NC, miR-206 inhibitor or miR-206 inhibitor NC, and results showed that miR-206 significantly increased the caspase-3 activity in A549 and SK-MES-1 cell lysate, by approximately 12 and 8.9 folds increase than that of bank A549 and SK-MES-1 cells (Fig. 8E and 8F), respectively. However, loss of miR-206 by transfecting with miR-206 inhibitor remarkably reduced the caspase-3 activity in A549 and SK-MES-1 cell lysate, by approximately 60% and 52% than that of bank A549 and SK-MES-1 cells (Fig. 8E and 8F), respectively. In addition, we also use dual-staining of annexin/PI to further evaluate the positive role of miR-206 in apoptosis of A549 cells. We found that miR-206 significantly promoted apoptosis in A549 cells, while inhibition of miR-206 reversed the high level of apoptosis in comparison to miR-206 treated A549 cells (Figure 8G). Further, our results of flow cytometric analysis demonstrated that forced expression of miR-206 resulted in a ∼40-fold increase in apoptotic cell death of A549 cell (Fig. 8H and 8I), while the percentage of apoptotic cells induced by miR-206 was decreased to the basal level when the cells were treated with the specific miR-206 inhibitor (Fig. 8H and 8I). These results demonstrated that miR-206 indeed promoted apoptosis in A549 cells.

### MiR-206 suppresses tumor growth *in vivo*

**Figure 9:**
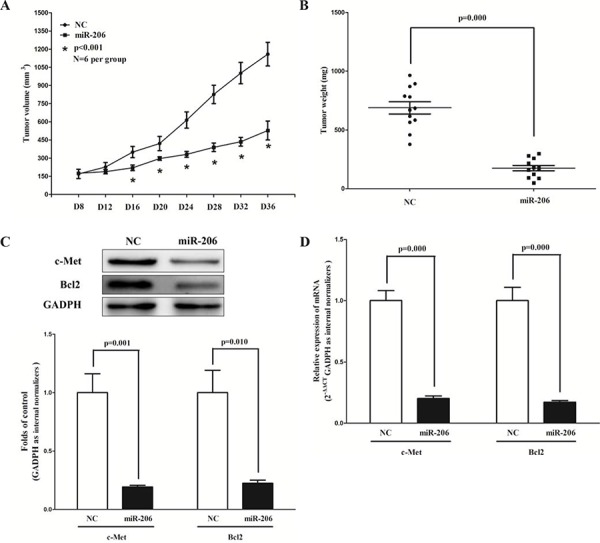
Ectopic expression of miR-206 suppresses tumor growth *in vivo* **A-B.** Tumor volume and weight in nude mice. Each group contained six mice (n = 6); the data are presented as the mean ± SEM; *p < 0.001, compared with the NC group. **C.** The expression of c-Met and Bcl2 protein in nude mice. **D.** The expression of c-Met and Bcl2 mRNA in nude mice. Assays were performed in triplicate. Means ± SEM are shown. Statistical analysis was conducted using student *t*-test.

To confirm the tumor suppressor role of miR-206 *in vivo*, we established a BALB/c nude mouse xenograft model using A549 cells. After 8 days, miR-206 agomir or miR agomir NC was directly injected into the implanted tumor every 4 days for seven times. The tumor volume was measured every 4 days until day 36. The tumor volume and weight of mice treated with miR-206 agomir were significantly reduced relative to that of miR agomir NC (Fig. 9A and 9B). This result indicates that miR-206 significantly inhibits the tumorigenicity of A549 cells in the nude mouse xenograft model. In addition, our results of western-blot and qRT-PCR demonstrated that the decreased expression of c-Met and Bcl2 in tumors developed from miR-206-agomir-treated nude mice relative to control tumors (Fig. 9C and 9D).

## DISCUSSION

Although dysregulation of miRNAs was reported in various types of human cancers [29], aberrant expression and potential role of miRNAs in lung cancers were under studied. MiR-206, which is highly expressed in human skeletal muscle, is down-regulated in many cancers. Evidence of miR-206 as a tumor growth suppressor has been reported in several cancers: miR-206 was found down-regulated in ERα-positive human breast cancer tissues and transfection of miR-206 into MCF-7 breast cancer cells inhibits cell growth in a dose- and time-dependent manner [30]. MiR-206 also promoted myogenic differentiation and block tumor growth in xenografted mice by down-regulation of Met tyrosine-kinase receptor, the product of the MET proto-oncogene [31]. Moreover, Yan *et al* also found that loss of miR-206 could contribute to aberrant cell proliferation and migration, leading to rhabdomyosarcoma development by suppression of c-Met [28]. Further, MiR-206 expression decreased in ERα-positive endometrial endometrioid adenocarcinoma (EEC) and its over-expression inhibited ERα-dependent proliferation, impaired invasiveness and induced cell cycle arrest in ERα-positive EEC cell lines [32]. All these findings indicated that miR-206 may play a tumor suppressor role in various cancers.

In the present study, we found that miR-206 was dramatically down-regulated in lung cancer tissues compared with matched normal lung tissues, and it inhibited the tumorigenic potential of lung cancer cells by down-regulating oncogenic targets, such as MET and Bcl2. To our knowledge, this is the first report that reveals detail mechanism between loss of miR-206 and tumorigenic potential of lung cancer cells. This contention is based upon the following observations. First, miR-206 expression was significantly reduced in primary lung cancer. Second, miR-206 can directly regulate mRNA expression by targeting the 3′-UTR of MET and BCL2, and inhibit protein expression of cyclin D1 and gene expression of cyclin D1, cyclin D2 and matrix metalloproteinase 9 (MMP-9), while increased p57 gene expression in lung cancer cell (A549). Third, miR-206 can also suppress tumor growth *in vivo*. Finally, ectopic expression of miR-206 in the lung cancer cell line (A549) significantly reduced cell growth, migration, invasion and colony formation, and promoted cell apoptosis, whereas depletion of miR-206 remarkably promoted lung cancer cell (A549) growth, migration, invasion and colony formation, and inhibited cell apoptosis.

Some miRNAs also function as oncogenes or tumor suppressors, just as protein-coding genes [33]. The increased tumorigenesis is associated with global reduction of miRNA [34]. In fact, interactions between mRNA and miRNA are tightly regulated, and even a small change of these interactions may cause severe consequences to cell physiology [35]. Accordingly, alterations in the expression of target genes could lead to disease states. MET proto-oncogene is located on chromosome 7q21–31 and encodes the receptor tyrosine kinase MET. In several tumors, MET signaling pathway is aberrantly activated and represents one of the most important mechanisms of progression and invasiveness [36, 37]. Furthermore, aberrant MET signaling activation has been identified as a prognostic factor of poor outcome in different solid tumors and also in lung cancers [26, 38–40]. BCL2, encoding the pro-survival protein Bcl2, is aberrantly activated in many tumors. In hematologic malignancies, BCL2 impairs cell apoptosis by overexpression of the pro-survival protein Bcl2 [41]. Because abnormally high levels of BCL2 sustain these tumors, there has been much interest in targeting BCL2 as a novel approach to treat lung cancer. Silence of MET and BCL2 expression inhibits lung cancer cell (A549 and SK-MES-1) growth, migration, invasion and apoptosis (Fig. 3A-3G). Our study showed that exogenous miR-206 could down-regulate the expression of c-Met and Bcl2 protein and mRNA in lung cancer cells. Moreover, the luciferase assay using a reporter containing the wild type miR-206 binding sequence at the 3′-UTR of c-Met and Bcl2 mRNA indicated that the luciferase activity could be significantly reduced or increased by over-expression or down-regulation of miR-206, which was in accordance with Yan's investigation that miR-206 targeted MET in Rhabdomyosarcoma. Furthermore, the c-Met and Bcl2 mRNA and protein were over-expressed in lung cancer tissues compared with normal tissues. Collectively, we discovered miR-206 targets 3′-UTR of c-Met and Bcl2 mRNA, and inhibits the expression of c-Met and Bcl2 in lung cancer cell lines (A549 and SK-MES-1).

We further showed that decrease in miR-206 expression is associated with an increase in oncogene CCND1, CCND2 and MMP-9, and a decrease in p57. CCND1 and CCND2 are well-established oncogenes in many different cancers [42, 43]. It has been reported that cyclin D1 is direct targets of miR-206 in 3T3-L1 cells [44] and HeLa cells [45], and over-expression of cyclin D1 promoted of breast cancer [44] and A549 [20] cells proliferation. Zhang *et al* revealed that cyclin D2 is also a directive target of miR-206 in gastric cancer (SGC-7901 cells) [46]. In accordance with these results, our study showed that cyclin D1 and cyclin D2 are also up-regulated in the majority of human primary lung tumors and cancer cell line (A549 and SK-MES-1). MMP-9, which belongs to a family of zinc-dependent endopeptidases, is collectively capable of degrading essentially all of the components of the extracellular matrix (ECM), and is considered to be associated with invasion and migration of tumor cell [47, 48]. Our data support the notion that the loss of miR-206 is associated with the up-regulation of MMP-9 level in lung cancer cells (A549 and SK-MES-1). p57, a cyclin-dependent kinase inhibitor, is considered to be a candidate of tumor suppressor gene that has been implicated in cancers [49]. Our study revealed that the over-expression of miR-206 is a associated with the up-regulation of p57 level in lung cancer cell (A549). These results are in accordance with Liu's research, they also discovered over-expression of miR-206 decreased MMP-9, and increased p57 expression, which leading to inhibition of Hepatocellular carcinoma (HCC) HepG2 cells invasion and proliferation [27].

Then we examined the mechanism of miR-206 on lung cancer cell growth, and found that over-expression of miR-206 significantly inhibited A549 cell proliferation, while loss of miR-206 promoted cell growth in A549 cells. Further, miR-206 also suppresses tumor growth *in vivo*. The growth-inhibition role of miR-206 may attribute to that miR-206 targets 3′-UTR of MET mRNA, and inhibits the expression of MET in lung cancer cells. In addition, miR-206 also inhibited CCND1 and CCND2 and increased p57 expression levels in lung cancer cells, which further contributed to the growth-delay efficacy of miR-206.

The present study also has shed light on the potential role of miR-206 on cancer metastasis. Metastasis is an integral part of tumor progression, and is a complex process attributed to loss of cellular adhesion, increased motility and invasiveness, entry and survival into the circulation, exit into new tissue, and eventual colonization of a distant site [50]. We showed that ectopic expression of miR-206 not only inhibited proliferation but also reduced cell migration and invasion motility of A549 cells. MET is a receptor for hepatocyte growth factor and a tyrosine kinase (receptor-type tyrosine kinase), and supports the initial steps of invasion and metastasis of most human cancers, including lung cancer [51]. The down-regulation of MET could be a possible mechanism by which miR-206 regulates growth and metastatic potential of these cells. Moreover, miR-206 also inhibited the expression of MMP-9, which was the key enzyme to promote tumor cell to invasion and migration.

We further investigated the role of miR-206 on lung cancer cell (A549) apoptosis. Apoptosis is a stereotypical process of cell death intrinsic to all multicellular eukaryotic organisms and is critical for the elimination of unwanted, infected, or otherwise damaged cells [52]. The effectors of this process are caspases, proteolytic enzymes that drive cellular demolition within the cell. Liu *et al* revealed that miR-206 over-expression inhibited the expression of Bcl2, but they did not further investigate the directed relationship between miR-206 and Bcl2. In our present study, we initially demonstrated that miR-206 promoted lung cancer cell (A549) death, by targeting 3′-UTR of BCL2, which encoded pro-survive protein Bcl2, and increased the casepase-3 activities.

Although Wang *et al* find in their study that miR-206 inhibits invasion and metastasis of lung cancer, they fail to investigate the potential mechanism [53]. Our study has investigated the whole role of miR-206 on lung cancer cell growth, migration, invasion, colony formation and apoptosis, and further examined the potential mechanism of miR-206 on lung cancer development.

In summary, we have shown that miR-206 is dramatically down-regulated in human lung cancer tissues compared with normal lung tissues. Moreover, up-regulation of miR-206 suppresses lung cancer cell migration, invasion and colony formation, and promotes lung cancer cell apoptosis, through targeting c-Met and Bcl2. In addition, miR-206 suppresses tumor cell growth *in vitro* and tumorigenicity *in vivo*. Collectively, our experimental data may provide a strategy for targeting the miR-206/c-Met or miR-206/Bcl2 interaction in a novel therapeutic application to treat lung cancer patients.

## Abbreviations

The abbreviations used are: used:

miR: microRNA
miR-206: has-microRNA-206
NSCLS: non-small cell lung cancer
3′-UTR: 3′-untranslated region
ERα: estrogen receptor alpha
BCL2: B-cell lymphoma-2
